# Electricity generation from food wastes and spent animal beddings with nutrients recirculation in catalytic fuel cell

**DOI:** 10.1038/s41598-020-67356-0

**Published:** 2020-07-01

**Authors:** S. O. Dahunsi

**Affiliations:** Sustainable Management of Natural Resources and Environment Research Group, Faculty of Environment and Labour Safety, Ton Duc Thang University, Ho Chi Minh City, Vietnam

**Keywords:** Biotechnology, Energy science and technology

## Abstract

A biochemical system was used for electricity generation from food waste (FW) and spent animal beddings (SAB). The wastes were blended and fermented anaerobically to produce fermentation liquids used as fuels for running a catalytic fuel cell. The fermentation liquids were analyzed for their components. The results show the organic contents i.e. volatile solids of both FW and SAB to be 23.4 and 20.9 g/L while the carbon contents were 6.5 and 6.1 g/L respectively. The media were however very rich in volatile fatty acids (VFAs). When used, the fermentation liquids from FW and SAB generated mean open-circuit voltages of 0.64 and 0.53 V and mean maximum power densities (*P*_mean_) of 1.6 and 1.3 mW/cm^2^ respectively. The fuel cell showed very high efficiency in the conversion of all VFAs especially butyric acid with the highest been 97% for FW and 96% for SAB.

## Introduction

The socio-economic growth and wellbeing of any nation depend largely on many factors chief among which is adequate power generation, effective distribution and efficient usage^1^. This explains why many nations of the world especially the developing ones are facing added dilemma in energy availability and this can be linked to factors such as lack of funds, low generating capacity, lack of continuity in leadership, poor investment policies, grid theft and sabotage, poor technical know-how, unnecessary bureaucracy and lack of efficient waste/environmental management policies. As a matter of fact, these factors have created a major gap between the demands and supply and the aftermath is the poor standard of living for the citizenry^2^. The International Energy Agency^3^ has estimated that well over 1.1 billion persons which make approximately 17% of the global population do not have access to electrical energy. Majority of these people lives in the countryside or very remote areas where several efforts at international, national and local levels which were geared towards the provision of energy supply has failed so miserably most of the time^4^. Besides, most of these affected localities have a very small population which further makes it difficult to provide energy. However, a holistic view of the aforementioned situations has created the impetus to invest in alternative sources of energy^5^. Such sustainable energies bring the added advantage of reduction in the annual emission of greenhouse gas (GHG), saving of cost on energy/fuel purchase by individuals, as well as in the enhancement in energy efficiency^6^.

Even though fossil fuels form the bedrock of most economies in the world, a major factor that limits the reliability and investment into this sector is the constant sudden increase in the price of fuels and other allied products. Again, this has created an opportunity for renewable energies to favorably compete in the energy market and possess the potentials to solve the power outage problems usually experienced especially in remote areas. As a result, several alternative energy devices have been developed and used. These include biogas cookers and lightning systems, diesel generator, micro turbines, photovoltaic apparatus, wind turbine generators, electric storage devices and fuel cells which can be fired by either natural gas or biomass. All these devices can be efficiently utilized irrespective of the location and time^7,8^. Among these methods, however, anaerobic digestion (AD) or fermentation of organic materials and biomass stands out as one of the most sustainable technology for the provision of green energy^9–16^.

According to the Food and Agricultural Organization^17^, well over 1.3 billion tons of food waste (FW) which represents a third of the global food production is churned out annually. In fact, both developed and developing nations have between 15 and 63% of their municipal waste streams to be FW^18,19^. In the same way, the world has doubled its total energy consumption over the last two decades most especially due to population surges in many countries^20^. Food waste has been ranked as number 3 out of 15 bioresources that provides major opportunities for productivity and investment^21^. This makes FW a profound substrate for energy generation and a sure way to address the ever-increasing challenges of food waste management especially in urban areas^22–25^. Due to FW’s richness in nutrients and biodegradable organic matter^26^, it has been thermally treated and processed to animal feed^27^, used for fertilizer production^28,29^, and energy generation via AD^30^. Besides, AD is one of the most promising technologies for efficient energy recovery from food waste by utilizing functional microbes for the conversion of the organic portion of FW into biogas with about 50 to 70% methane content^31–33^.

However, the major challenge in the AD of FW is the high volatile fatty acids (VFAs) accumulation leading to enormous acid production^26,34^ and this is due to the easy biodegradability of FW besides its high carbon-to-nitrogen (C/N) ratio^35,36^. As a result of these factors, FW is quickly hydrolyzed to VFAs but takes a lot of time for the subsequent conversion to methane^34^, hence the methanogenesis stage is rate-limiting in the AD of FW. As this happens, VFAs are accumulated and the system more acidified as evidenced in pH decrease. When this is prolonged, it can cause inhibition to the activities of functional microbes and slow down the rate of methane generation or completely halt the digestion process.

Spent animal beddings (SAB) are solid wastes usually accumulated in deep-litter systems where materials like straw, sawdust, and other biomass are used as an absorbent for animal excrements and urine. They accumulate in the stables, ranches and experimental animal houses^37^. It has been reported that 53.8% of spent animal beddings has a total solids (TS) contents of 18% and above^38^ making them suitable materials for AD. Besides, the properties of SAB are hardly or sparsely described in the literature as against those of manure and straw separately whereas the beddings is a profound energy substrate having had its properties modified by passing through the animal’s mechanical action on the litter coupled with the biological degradation during litter accumulation^37^. However, the bulk of spent animal beddings are FW accrued from the animal's feeding and remnants from the feeds. It is, therefore, a waste stream with similar properties to FW especially with the fact that the animal feeds are also composed of proteins, carbohydrates, and lipids which are the major components of human food.

Considering the inherent properties of both FW and SAB and the opportunities that abound in the conversion of these wastes to energy, it is expedient to seek an alternative treatment system that will physically separate the fermentation and the methanogenesis stages in order to avoid the rate-limiting phenomenon during methanogenesis^39^. In this way, it can be possible to use the fermentation products i.e. VFAs for direct energy production without necessarily progressing to methanogenesis. This can be possible by replacing the slow biological methanogenesis pathway with a rapid chemical process which will directly and efficiently convert fermentation products into energy. Prior to this time, Xu et al.^40^ carried out an experiment in which a liquid catalytic fuel cell utilizing different organic substrates as fuel was developed and used. In actual operation, the organic substances are rapidly converted to carbon dioxide and water during which electricity is simultaneously generated. The advantages of this system are adaptation to impurities long term stability^41^. Earlier, a fuel cell produced up to 49.8 mW/cm^2^ at 100 °C when phosphomolybdic acid was used as a catalyst while the fuel was sewage sludge^42^.

It is therefore proposed in this study to use a catalytic fuel cell to directly convert fermentation products from FW and SAB into electricity. This going by existing literature is the very first attempt to explore this treatment for these two abundant waste streams. The advantage of this biological fermentation and chemical conversion is high efficiency, stability and elimination of inhibition that would have occurred to methanogens. Even though some previous researchers have studied the anaerobic fermentation (AnF) of FW, several intermediate acids^43^, ethanol^44^, lactic acid^45^, and butanol^46^ were the targets and the rate of degradation varied according to the different fermentation types^45^. Though, SAB had been previously utilized for energy generation in some studies^37,47–50^, the treatment method employed in all these experiments was AD in which all the four processes of digestion i.e. hydrolysis, acidogenesis, acetogenesis and methanogenesis were explored using different anaerobic digester designs/configurations with the target product been biomethane. In this study however, the AnF of SAB is being studied for the first time as a waste with similar characteristics to FW and with the sole aim of generating fermentation liquids for running a fuel cell. Besides, all the previous studies utilized SAB from livestock houses (Piggery, dairy etc.) while this study focused on SAB from experimental animals (Rats, mice, rabbits and other guinea pigs). The aim of this study, therefore, is to evaluate the electricity generation from the AnF of FW and SAB using a fuel cell for the conversion of the produced fermentation products. This is expected to clearly demonstrate the efficiency of the anaerobic fermentation-fuel cell (AnF-FC) synergy and design future applications.

## Materials and methods

### Collection of materials

The FW used was collected from a University cafeteria and it was a mixture of main carbohydrates including cooked rice, yam, bread, maize, wheat and other food grades like beef, beans, vegetables, and condiments/seasonings. The SAB, on the other hand, was collected from the animal house where experimental animals (Rats, mice, rabbits and other guinea pigs) are kept under the Biochemistry Department of the University. All the food material was blended together using a laboratory blender to constitute the FW that was used for feeding the reactor. The SAB which was more of animal food waste, droppings and shavings were also blended before further use.

### Description of the fuel cell, catalysts, and fuels

The fuel cell used in this study is very similar to the one earlier described^40^. The structure consists of two chambers i.e. an anode and a cathode in an acrylonitrile butadiene styrene plastic container. The two chambers were divided by a Nafion 115 proton-exchange membrane (Dupont, USA). The electrode had a diameter of 10 mm × 20 mm × 20 mm and was made of graphite felt filling each of the two chambers alongside a carbon plate for the collection of electric current. By the sidewall of each of the chambers were an inlet for injection of electrolyte and an outlet for its outflow. In the analytic portion of the cell, FeCl_3_ and phosphomolybdic acid (H_3_PMo_12_O_40_, PMo_12_) (Zhanyun, Shanghai, China) were used as co-catalysts for the electro-oxidation of fuels and they have high potency on fuels such as glucose, starch, protein i.e. egg albumin and fermentation liquid (VFAs) as produced and utilized in this study. Lipid was not included since it has been established that fuel cells lack the capacity to degrade lipids^51^. However, the catalyst used in the catholyte was non-Keggin-type molybdovanadophosphoric acid (H_12_P_3_Mo_18_V7O_85_) earlier synthesized^52^. The choice of this catalyst was its high affinity for oxygen in the collection of electrons from cathodes.

### Anaerobic reactors

The computer controlled split-chain anaerobic reactors with continuous stirring with a working volume of 5 L, a temperature of 35 °C and retention time of 4 days were employed in the AnF of FW and SAB in order to generate the fermentation liquid. Before feeding into the reactors, the lipid components of both FW and SAB were extracted out using established protocols^53–55^ for the purpose of biodiesel production. The mixtures from FW and SAB were thereafter fed into the reactors once daily. Subsequently, the fermentation liquid was obtained by centrifuging the effluents from the FW and SAB reactors at 5,800 *g* and the supernatants were used as the fuels in the fuel cell.

### Operation of the fuel cell

The fuel cell used in this study was operated in two different modes i.e. batch and continuous. This was done to evaluate its performance in terms of discharge and also the degradation of the different fuels respectively^51^. The batch operation was used to serve as a 2 h pre-treatment prior to the continuous one that lasts 48 h. The formulation of the components of each mode was done separately and all analyses were carried out in triplicate and the average values were used for the fuel cell’s performance evaluation. In batch phase, the anolyte had a total volume of 25 mL consisting of 0.1-mol/L PMo_12_, 0.5-mol/L FeCl_3_, 5 mL of 85 wt% H_3_PO_4_, 180 g/L each of glucose and starch, 80 g/L protein, 40 g/L lipid, 800 mL/L fermentation liquid and deionized water. The compound was thereafter heated in a water bath at 95 °C for 2 h and maintained at 37 °C in the absence of light. For the catholyte, 0.3 mol/L molybdovanadophosphoric acid (H_12_P_3_Mo_18_V7O_85_) was used. In both cases, the anolyte and catholyte were made to circulate between the containing vessels and the fuel cell via a silicone pipe at a flow rate of 13.3 mL/min. The open circuit voltage of the fuel cell was then tested using a multimeter and the I–V curve by linear sweep voltammetry on a CHI 600E electrochemical workstation (Chenhua Instrument, China) when voltage stability was achieved. By this, there was a decrease to 0.01 V at a scanning speed of 0.1 mV/s and record of the current variations and power densities were taken.

**Figure 1:**
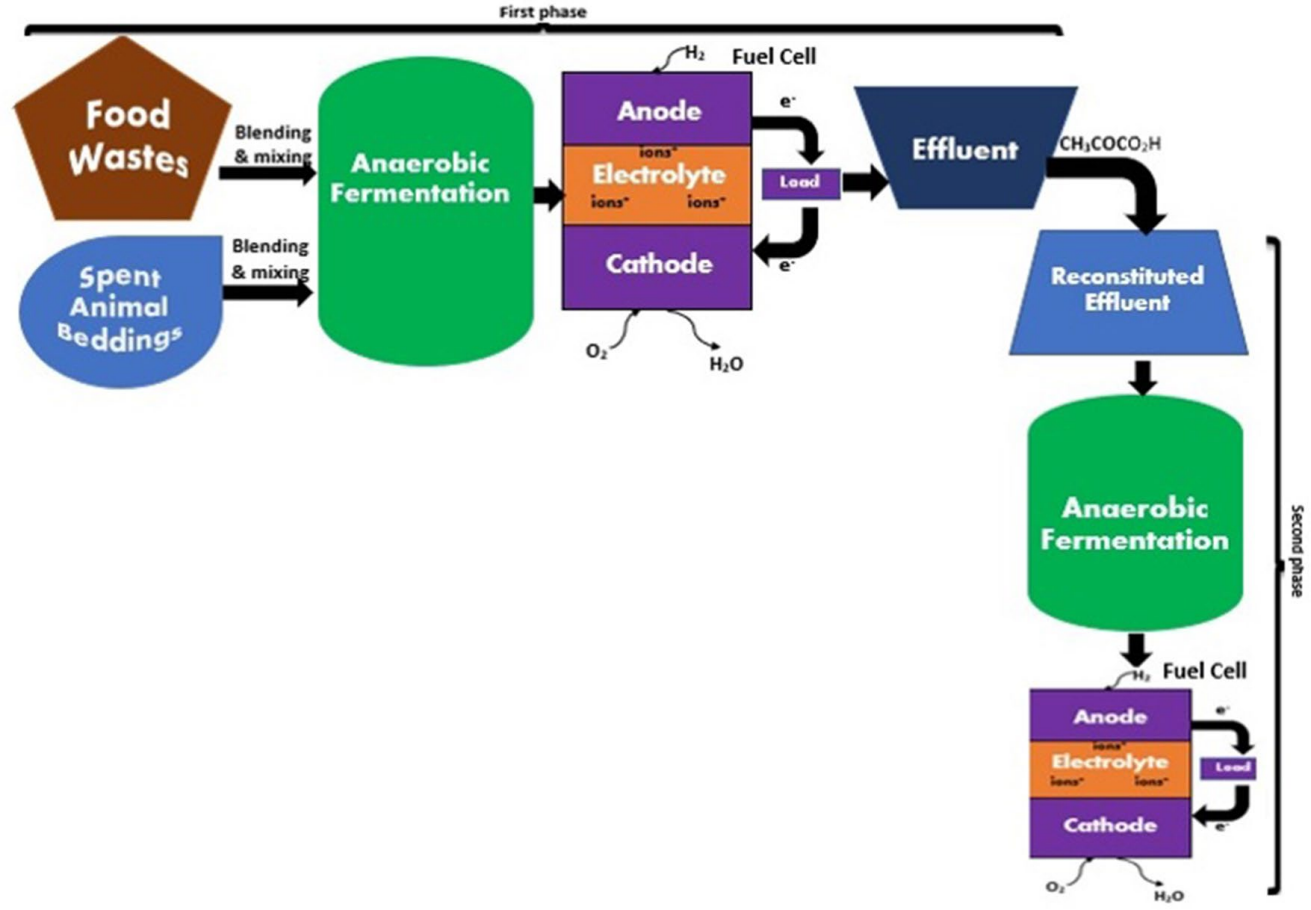
This shows the schematic diagram of the experimental set up showing both the first and second phases of experiment.

For the formulation of the continuous phase, the anolyte’s composition was 0.1 mol/L PMo_12_, 1.0 mol/L FeCl_3_, 5 mL of 85 wt% H_3_PO_4_, fermentation liquid and deionized water. As done for the batch formulation, this mixture was heated in the water bath at 95 °C for 2 h, allowed to cool before connecting to the fuel cell while the temperature was adjusted and maintained at 80 °C. The catholyte was simultaneously connected to the fuel cell while the electrolyte was continuously made to recycle between the cell and the container at 13.3 mL/min flow rate. After this, a rubber plug was used to seal the anolyte vessel and two different pipes for sampling gases and liquid were used to pierce the plug. An ice box was initially used for condensing the generated gas during anodic reactions before final collection with a sampling bag. The schematic diagram of the experimental set up is shown in Fig. 1

### Efficiency and energy recovery

In determining the overall bioelectrochemical performance and electrical energy recovery in this study, the temporal trend of the fuel cell electrical behavior for each continuous AnF was observed. In doing this, measurement of the external resistive circuit was taken into consideration after which the current density (The cathodic surface) and the power density (Total volume of reactor) were calculated. The total electric energy recovered (E_recovered_) in kJ/m^3^ from each fermentation liquid was determined by integrating measured electric power over each treatment time^56,57^. Since the batch reactors were fed with complex substrates, the calculation was based on the COD concentration COD of both FW and SAB. To begin with, the COD removal efficiency for each treatment was determined as follows:

1 $$\eta =\frac{\left({COD}_{in}-{COD}_{out}\right)}{{COD}_{in}}\times 100$$

where $\eta$ = *COD* removal efficiency, *COD*_in_ (g/L) = influent concentration (Before treatment), *COD*_out_ (g/L) = effluent concentration (After treatment).

In fuel cell’s operation, the hydraulic retention time (HRT) usually varies which requires the calculation of a normalized COD removal rate for comparison purposes especially between different substrates used in fuel cell experiments. The normalized *COD* removal rate (*NCRR*) was calculated in this study thus:

2 $$NCRR=\frac{\left({COD}_{in}-{COD}_{out}\right)}{HRT}\times 100$$

The electrical energy recovery was calculated using the formula below:

3 $${E}_{recovered}=\underset{{t}_{1}}{\overset{{t}_{2}}{\int }}{P}_{mean}dt$$

where ${t}_{1}$ = the initial continuous fermentation time, *t*_2_ = the final continuous fermentation time, ${P}_{mean}$ = the power density.

### Analyses

As shown in Table 1, various analyses were carried out on the fermentation fluid. Determination of total solids (TS), volatile solids (VS) and Chemical oxygen demand (COD) was carried out using the American Public Health Association’s method for the analysis of water and wastewaters^58^. Measurement of total organic carbon (TOC) and total nitrogen (TN) was done with the aid of a TOC analyzer (TOCL, Shimadzu, Kyoto, Japan). Concentration of volatile fatty acids (VFAs) were determined chromatographically (Clarus 580GC PerkinElmer, USA) to which a flame ionization detector was attached while that of total ammonia nitrogen (TAN) was carried out spectrophotometrically (Spec DR3900, HACH, USA) using Nessler's reagent with potassium sodium tartrate used for screening metal ions. Measurement of pH was by a pH meter (PHS-3C, Shanghai, China) while the nitrogen (N_2_) and carbon dioxide (CO_2_) obtained from the organic breakdown of substrates were analyzed using the 7850S gas chromatograph (Jinghe, Shanghai, China) which was equipped with a thermal conductive detector at 150 °C column temperature.

**Table 1:** Characteristics of fermentation liquid from food waste and spent animal beddings.

Parameters (g/L)	Food waste	Spent animal beddings
Total solids (TS)	38.5 ± 0.02^a^	41.2 ± 3.00^b^
Volatile solids (VS)	23.40 ± 0.01^a^	20.90 ± 2.02^a^
Total organic carbon (TOC)	6.50 ± 0.02^a^	6.10 ± 1.02^a^
Total nitrogen (TN)	0.23 ± 0.01^a^	0.24 ± 0.01^a^
Chemical oxygen demand (COD)	15.29 ± 0.02^a^	17.91 ± 2.12^b^
Acetic acid	10.02 ± 1.02^a^	8.99 ± 0.12^b^
Propionic acid	1.78 ± 0.02^a^	1.62 ± 0.02^a^
Butyric acid	1.33 ± 0.01^a^	1.27 ± 0.02^a^
Isobutyric acid	0.49 ± 0.01^a^	0.43 ± 0.01^a^
Valeric acid	1.66 ± 0.00^a^	1.50 ± 0.02^a^
Isovaleric acid	0.06 ± 0.01^a^	0.05 ± 0.01^a^
Ammonia	0.61 ± 0.01^a^	0.62 ± 0.01^a^

Values shown in table are means of triplicate analyses with respective standard errors; superscripts with same letters are statistically the same by the Tukey’s test at 5%.

## Results and discussion

### Performance of fuel cell

Table 1 shows the characteristics of both fermentation media. Before the actual continuous experiment, the batch treatment was done by butyric fermentation with acetic acid as the major product based on the suggestion of Liu et al.^51^ when a very high rate of acetic acid conversion was reported when the fermentation liquid from the FW treatment was used in a fuel cell. In this study, the fermentation liquids from FW and SAB generated mean open-circuit voltages of 0.64 and 0.53 V and *P*_mean_ of 1.6 and 1.3 mW/cm^2^ respectively.. In reality, FW fuel has a higher power density than the value (1.2 mW/cm^2^) obtained by Liu et al.^51^ but lower than the 3.4 mW/cm^2^ reported by Xu et al.^40^ when glucose was used as fuel. Interestingly, SAB fuel also performed better than the earlier report for FW^51^. The reason for these observations could be directly due to the relatively high organic load of the two fermentation liquids in this study i.e. 23.4 and 20.9 g/L for FW and SAB respectively^59,60^. However, these are still far lower than the 180 g/L glucose concentration used in the study of Xu et al.^40^ which yielded a better result. In the use of fermentation liquid for fuel cell therefore, a higher concentration of the liquid is required to enhance the performance efficiency. Since the rate of converting substrates to fermentation fluid during anaerobic fermentation depends on several factors among which are the organic loading rate (OLR) and pH^43,61^, these two factors were strictly regulated in this study in order to achieve higher working efficiency of the fuel cell. The reactors were dosed with sodium hydroxide (NaOH) in order to stabilize the pH at between 6.0–6.5 for both reactors and this gives a hydrolysis rate of 62 and 50% for FW and SAB respectively. These results are very similar to those obtained by Wu et al.^62^ when a rate of 60% was reported for FW at the working pH of 6.0. At this condition, the major products of the fermentation were VFAs as against lactic acids and ethanol which would have dominated the process had lower pH values been used^62,63^. The predominant among the VFAs were acetic and butyric acids and this justifies the initial use of butyric fermentation at a pH of 6.2 as the pre-positive treatment before the real experiment.

**Figure 2:**
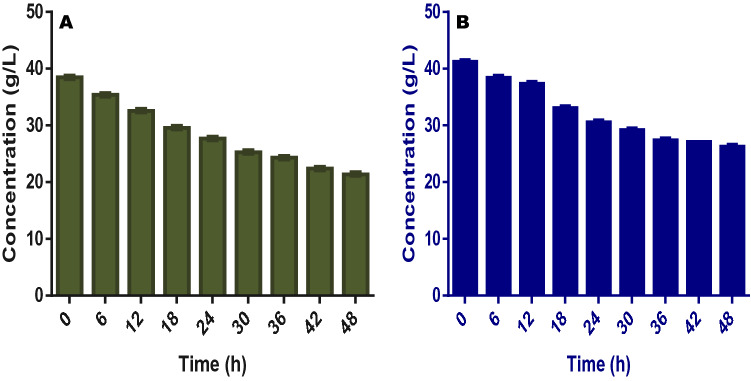
This figure shows the pattern of total solids (TS) conversion for (**A**) food wastes and (**B**) spent animal beddings during the experiments.

In terms of nutrients degradation and utilization, it was obvious that the fuel cell was able to efficiently utilize the nutrients in the anolyte and this was done within a short experimental period. As shown in Table 2, TS reduced from the initial concentration of 38.5 and 41.2 g/L to 21.4 and 26.3 g/L for FW and SAB respectively after the cell stopped electricity production. For VS degradation which represents the utilization of the organic component of the fermentation liquid, the initial concentration of 23.4 and 20.9 g/L for FW and SAB were degraded to 4.7 and 5.1 g/L respectively. These show 80 and 76% reductions for both FW and SAB at the end of the 48 h of the experiment (Fig. 2).

**Table 2:** Characteristics of effluent from food waste and spent animal beddings treatment in fuel cells (first phase).

Parameters (g/L)	Food waste	Spent animal beddings
Total solids (TS)	21.41 ± 3.02^a^	26.6 ± 3.02^b^
Volatile solids (VS)	4.70 ± 1.02^a^	5.10 ± 1.02^a^
Total organic carbon (TOC)	1.10 ± 0.01^a^	1.40 ± 0.05^b^
Total nitrogen (TN)	0.03 ± 0.00^a^	0.04 ± 0.01^a^
Chemical oxygen demand (COD)	3.42 ± 0.02^a^	4.66 ± 0.12^b^
Acetic acid	1.01 ± 0.01^a^	1.04 ± 0.02^a^
Propionic acid	0.23 ± 0.01^a^	0.17 ± 0.02^b^
Butyric acid	0.04 ± 0.01^a^	0.05 ± 0.02^a^
Isobutyric acid	0.03 ± 0.00^a^	0.03 ± 0.01^a^
Valeric acid	0.82 ± 0.01^a^	0.23 ± 0.01^b^
Isovaleric acid	0.01 ± 0.01^a^	0.01 ± 0.01^a^
Ammonia	0.23 ± 0.03^a^	0.19 ± 0.01^b^

Values shown in table are means of triplicate analyses with respective standard errors; superscripts with same letters are statistically the same by the Tukey’s test at 5%.

**Figure 3:**
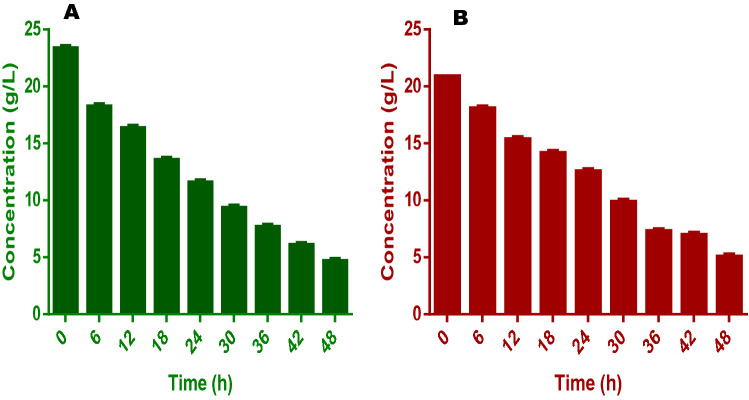
This figure shows the pattern of volatile solids (VS) conversion for (**A**) food wastes and (**B**) spent animal beddings during the experiments.

An interesting pattern of degradation was equally shown by the fuel cell in that there were only 23 and 20% degradation of VS in both FW and SAB after the first 12 h and this progressed to 56 and 49% respectively at the end of 24 h. This suggests a steady and continuous utilization of the organic substances as the experiment progressed. In the long run, the fermentation liquid from FW showed better nutrient utilization than that of the SAB when used in the fuel cell (Fig. 3).

**Figure 4:**
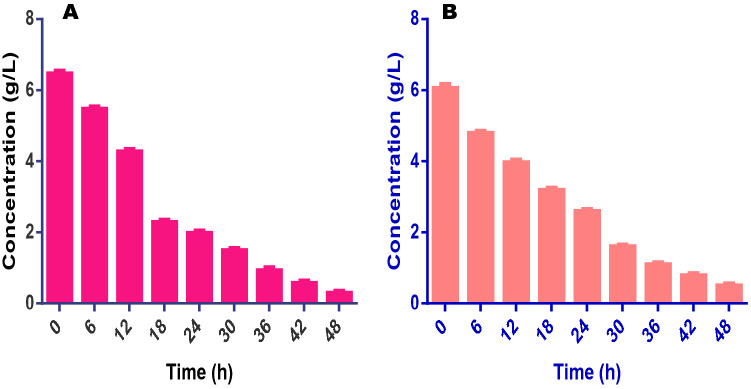
This figure shows the pattern of total organic carbon (TOC) conversion for (**A**) food wastes and (**B**) spent animal beddings during the experiments.

Among all the factors responsible for the efficiency of a fuel cell in treating fermentation liquid is the composition and oxidation pattern of organic carbon (TOC). In this study, when the fuel cell was operated in continuous mode, there was a rapid degradation of TOC. The TOC values at the end of 12 h operation were 4.3 and 4.5 g/L from the initial concentrations of 6.5 and 6.1 g/L for FW and AB. After this, degradation was slower and picked up again at about the 25 h of the experiment. At the end of 48 h, the final values of TOC in the fuel cells treating FW and SAB were 0.3 and 0.5 g/L respectively (Fig. 4). These mean that at the end of the experiments, 95 and 92% of the initial TOC in FW and SAB fermentation liquids had been utilized. It was also noticed that bulk of the TOC in the liquids was drawn from the VFAs which is evident in the high diversity and concentrations of the VFAs in this study. This phenomenon also impacted greatly on the degradation or conversion of the VFA for the efficiency of the cells in both FW and SAB experiments.

**Figure 5:**
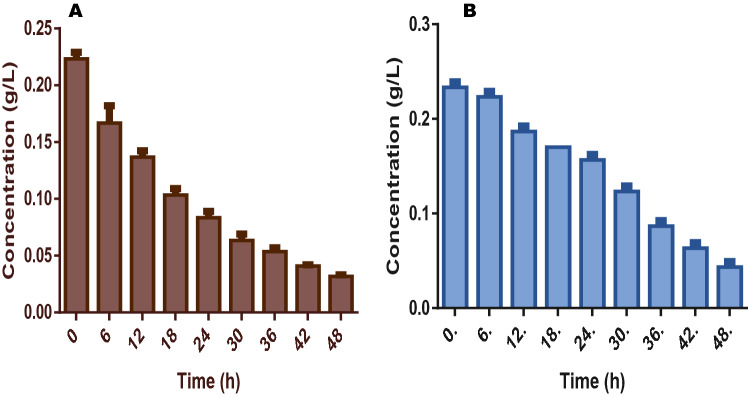
This figure shows the pattern of total nitrogen (TN) conversion for (**A**) food wastes and (**B**) spent animal beddings during the experiments.

This efficient conversion of organic carbon had a great impact on the electricity generation by the cells. At about the 12 h of the experiment, most organic substances in the liquids were just being degraded to different other degradable forms and the cells at this point showed a decrease in voltage from 0.48 to 0.07 and 0.11 V for FW and SAB. This decrease in voltage was observed to continue steadily and was almost at zero in both fuel cells by the end of the experiments. This simply means that there all the earlier formed intermediate substances were degraded though slowly. Similar to the degradation pattern of TOC, the concentration of total nitrogen (TN) followed the same trend. In the FW fermentation liquid, the concentration reduced from 0.23 to 0.03 g/L at the end of 48 h which translate to 87% reduction while in the SAB fermentation liquid, a final 83% reduction was observed i.e. from initial 0.24 g/L to a final of 0.04 g/L. In an earlier study^42^, had reported the two major possibilities for maximum TN reduction which are converted to nitrogen gas (N_2_) and binding to PMo12 in order to form ammonium phosphomolybdate (APM) which is most unlikely considering the low concentration of TN in the medium before treatment in fuel cells. Even in the likelihood of a combination of nitrogen and APM, the latter can still be efficiently used as a catalyst after dissolution in a solution of phosphoric acid. This still gives rooms for speculations as to the final fate of nitrogen during the fuel cell experiments involving FW and SAB fermentation liquid. As for ammonia which is usually formed from the degradation of the protein, its concentration was higher at the beginning due to its protein degradation giving rise to ammonia formation. It was finally converted to nitrogen gas and CO_2_ after experiencing a slow degradation with slight accumulation towards the end of the experiment. In FW fermentation liquid, ammonia degraded from initial concentration of 0.61 g/L to 0.23 g/L at 48 h representing 62% degradation/conversion. For SAB, there was 69% degradation i.e. from 0.62 to 0.19 g/L as shown in Fig. 5.

**Figure 6:**
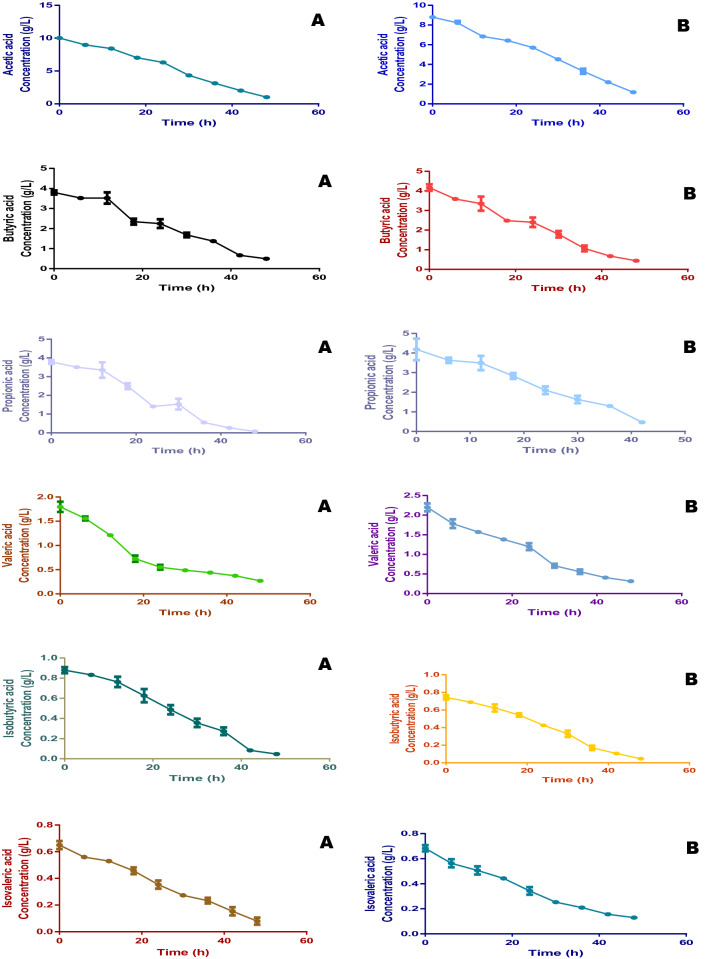
This figure shows the pattern of volatile fatty acids (VFAs) conversion for (**A**) food wastes and (**B**) spent animal beddings during the experiments.

The efficiency of the fuels cells to utilize VFAs as fuel for electricity generation was the major objective of this study. In the assessment of VFAs utilization, therefore, it was discovered that virtually all VFAs present in the fermentation liquids were efficiently converted to CO_2_ and other important molecules which contributed to the high performance seen in the fuel cells. The high conversion rate of VFAs seen in this study was made possible by the actions of the co-catalysts (FeCl_3_ and phosphomolybdic acid (H_3_PMo_12_O_40_, PMo_12_)) which brought about the oxidation of the VFAs. As shown in Tables 1 and 2, VFAs detected in the fermentation liquids produced from FW and SAB includes the three major VFAs acetic, propionic and butyric and others such as valeric, isobutyric and isovaleric acids. In both FW and SAB, acetic acid dominated the total VFAs (TVFAs) composition accounting for 66.3 and 65% of TVFAs in each case. Propionic and butyric acids were the second most abundant with 20 and 21% in FW and SAB respectively. Following these major acids is valeric acid having a composition of 11% in both media while others are isobutyric and isovaleric acids. The VFAs profile of both FW and SAB in this study provides a considerable platform for comparing both materials in the production of fermentation medium since they have a very similar and almost equal composition of VFAs. The initial concentration of acetic acid in FW medium was 10.02 g/L which decreased significantly with treatment time, an indication of efficient degradation with a final concentration of 1.01 g/L i.e. 90% degradation. For SAB, acetic acid decreased from 8.99 to 1.04 g/L which account for 88.4% degradation. Overall, the degradation levels of propionic, butyric, valeric, isobutyric and isovaleric acids in the fuel cell treating FW fermentation medium were 87.1, 97, 84, 94 and 83% respectively. Similarly, in the fuel cell treating SAB fermentation liquid, the degradation of the same set of VFAs were 90, 96, 85, 93 and 80% respectively after the 48 h of treatment (Fig. 6). A major inference here is that fuel cells treating fermentation liquids are capable of efficiently utilizing smaller molecules.

**Figure 7:**
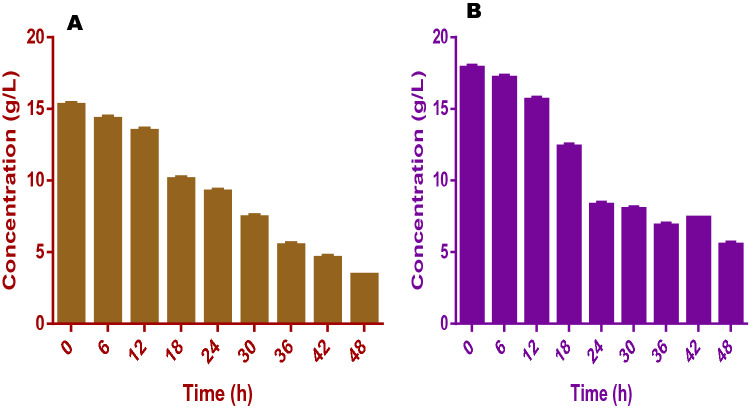
This figure shows the pattern of total soluble chemical oxygen demand (SCOD) conversion for (**A**) food wastes and (**B**) spent animal beddings during the experiments.

Evidence of further organic matter mineralization was seen in the decrease in the concentration of soluble chemical oxygen demand (SCOD) in both fuel cells all through the treatment period. This further supports the assertion that VFAs were broken down to CO_2_, H_2_O, and other small organic molecules. In the fuel cell treating FW fermentation liquid, SCOD decreased by 78% i.e. from initial 15.29 to a final concentration of 3.42 g/L at 48 h treatment time (Fig. 7). Also, in the SAB fermentation liquid treatment cell, the final value of SCOD was 5.56 g/L as against the initial value of 17.91 g/L which translate to a reduction of 74% after 48 h. These results corroborate the earlier submission^19^ that biochemical reactions in a fuel cell can cause enormous decomposition of organic substances due to the provision of more reduced catalysts which in turn help in organic matter’s oxidation especially in the anolyte.

### Mono treatment of FW and SAB organic components

As earlier reported^40^, one way to confirm the efficiency of a fuel cell in converting the organic constituents of FW was to directly and singly treat the major components and this further showed the extent of degradation or utilization of each component in the fermentation liquid and the extent of contribution toward the overall electricity generation from the cell. Even though this was reported for FW in that previous study, there is a need to do the same for the major constituents of SAB which are not very different from those of FW. Another major observation here is that even the composition of FW differs according to the types of food materials coming into the stream. This also necessitated that the components in the FW used in this study be treated individually in a fuel cell. All these were carried without prior positive treatment before actual fuel cell reactions. In doing this, glucose, starch, and protein were each used as fuel in the cells and their respective degradation and input toward electricity generation were quantified. Lipid was not considered in this study since it was already extracted from the substrates and more importantly because it cannot be degraded in a fuel cell according to earlier reports owing to its insolubility and structure.

The use of glucose as fuel yielded very high power densities of 6.7 mW/cm^2^. This value is very similar but slightly higher than obtained via the use of glucose in previous studies^40,51^ which utilized the same co-catalysts as used in the current study. In the usage of starch as fuel in the cell, the power density reached 2.1 mW/cm^2^ which was far lower than the result obtained in the use of glucose since starch showed a lower level of catalytic reactions than glucose in the fuel cell. Besides, starch is known to gelatinize and this would possibly make the reaction medium more viscosity with the attendant effect of a lower transfer of mass and subsequent power generation. By using protein, the power density was 1.8 mW/cm^2^. This equally showed a slightly higher performance by starch since proteins are known to denature thereby forming several other compounds whose presence and concentration could cause the reaction medium to also become viscous and this ultimately slow down the rate of reaction and could even lead to no reaction at all. Another reason that could cause a better performance of starch over protein is the composition of starch in terms of the hydroxyls and this aided the reaction process with evidence of higher electricity generation. This phenomenon had earlier reported^64^. The summation of this is that glucose remained the most efficient in fuel cell experiment which is easily linked to the far higher organic constituent in glucose than most fermentation liquids ever reported. This calls for the use of liquid fuels with higher organic loading as this will definitely enhance the efficacy of electricity generation from fuel cells. In themselves, fermentation liquids are highly potent as fuels considering their compositions of different organic materials, acids and nutrient elements than ordinary glucose. Therefore, fermentation liquids should be used with higher organic constituents in order to enhance their discharge performance in fuel cells.

It is noteworthy to say that there may still be some lipid residues in the substrates used in this study owing to the fact that lipids are usually not 100% removed from food substances especially those that are lipid-rich. These residues must have been dealt with in the 2 h batch treatment and subsequently converted to simple VFAs and this explain why no lipid clogging effect was reported in this study. It is, therefore, necessary to always remove the lipid portion of FW and even SAB as seen in this study and to also pretreat such substances in short batch experiments prior to real continuous treatment in fuel cells. The extracted lipid is not usually wasted as it would make a very good candidate for the biodiesel production via catalytic transesterification and esterification reactions.

### Nutrient recirculation

The effluents from the reactors treating both FW and SAB fermentation liquids were closely examined after the continuous experiments to see the possibility of re-use especially since they still contain some levels of nutrients. Even though the VFAs in these liquids have been almost completely used up in the generation of electricity by the fuel cells, there is still a considerable composition of VS and SCOD which are the main sources of organic matter. Though TOC was efficiently used up during continuous treatment, a boost in the organic carbon via external source can make the liquids resuscitated thereby energizing the bacterial community in using up the carbon and converting the organic matter to VFAs. By so doing, a new and viable fermentation liquid can be reconstituted. In this study, both effluents were dosed with 20, 30, 40 and 50 g/L pyruvate (CH_3_COCO_2_H) as external carbon source and fed into the anaerobic reactors for a 4-day fermentation as earlier done for the FW and SAB. The characteristics of the fermentation media resulting from these are shown in Table 3 and they were then used in the fuel cells for electricity generation for another 48 h. Results in the table showed that the addition of pyruvate energized the microbial communities in degrading the VS, SCOD, and TOC evident by the formation of some levels of all the VFAs earlier reported in the media. Also, highest *P*_mean_ of 0.8 and 0.5 mW/cm^2^ for FW and SAB respectively were reported in this second phase of power generation. Thus, this study has shown the high potential of VFAs regeneration and additional electricity production by the addition of an external carbon source to already used fermentation medium.

**Table 3:** Characteristics of fermentation liquid from food waste and spent animal beddings after reconstitution.

Parameters (g/L)	Food waste	Spent animal beddings
Total solids (TS)	21.2 ± 2.02^a^	27.1 ± 3.01^b^
Volatile solids (VS)	4.83 ± 0.01^a^	5.60 ± 0.01^a^
Total organic carbon (TOC)	1.30 ± 0.01^a^	2.20 ± 0.01^b^
Total nitrogen (TN)	0.21 ± 0.00	0.06 ± 0.00
Chemical oxygen demand (COD)	3.94 ± 0.11^a^	4.88 ± 0.03^b^
Acetic acid	4.51 ± 0.01^a^	4.06 ± 0.03^b^
Propionic acid	1.03 ± 0.03^a^	1.00 ± 0.01^a^
Butyric acid	0.94 ± 0.02^a^	0.55 ± 0.03^b^
Isobutyric acid	0.44 ± 0.02^a^	0.35 ± 0.02^a^
Valeric acid	1.02 ± 0.01^a^	1.01 ± 0.01^a^
Isovaleric acid	0.04 ± 0.01^a^	0.02 ± 0.01^a^
Ammonia	0.13 ± 0.01^a^	0.11 ± 0.02^a^

Values shown in table are means of triplicate analyses with respective standard errors; superscripts with same letters are statistically the same by the Tukey’s test at 5%.

The characteristics of the final effluents after the second treatment (Table 4) showed that they can be used as fertilizers for crop plants sustainable wellbeing in many cropping systems. This is most possible because the effluents are rich in suitable microbial inoculants as well as easily degraded fibers.

**Table 4:** Characteristics of effluent from food waste and spent animal beddings treatment in fuel cells (second phase).

Parameters (g/L)	Food waste	Spent animal beddings
Total solids (TS)	13.11 ± 3.02^a^	16.1 ± 1.02^b^
Volatile solids (VS)	1.39 ± 1.02^a^	1.70 ± 1.02^b^
Total organic carbon (TOC)	1.06 ± 0.01^a^	0.80 ± 0.05^b^
Total nitrogen (TN)	0.03 ± 0.00^a^	0.01 ± 0.01^a^
Chemical oxygen demand (COD)	1.72 ± 0.02^a^	2.24 ± 0.10^b^
Acetic acid	1.71 ± 0.01^a^	1.44 ± 0.02^a^
Propionic acid	0.03 ± 0.01^a^	0.07 ± 0.02^b^
Butyric acid	0.15 ± 0.01^a^	0.03 ± 0.02^b^
Isobutyric acid	0.13 ± 0.00^a^	0.11 ± 0.01^a^
Valeric acid	0.21 ± 0.01^a^	0.00 ± 0.00
Isovaleric acid	0.00 ± 0.00	0.00 ± 0.00
Ammonia	0.03 ± 0.01^a^	0.03 ± 0.01^a^

Values shown in table are means of triplicate analyses with respective standard errors; superscripts with same letters are statistically the same by the Tukey’s test at 5%.

### Overall performance assessment of fuel cell

In this study, the overall treatment period was only 6 days i.e. 4 days for the batch AnF and 2 days in the continuous system. Thus, using a fuel cell is a more efficient way of energy generation with very shorter retention time than most conventional energy generation methods. Anaerobic digestion (AD) though is an efficient energy generation pathway, will require up to 30 days treatment in a controlled system or more in an uncontrolled system depending on ambient temperature. Also, a fuel cell as seen in this study was very efficient in organic matter degradation as up to 95% initial TOC was degraded while almost all VFAs that were reported in the media were consumed by the microbial community. This equally showed that a fuel cell provides better ambiance for acidogenic microbial activities which results in the maximal consumption of VFAs unlike in AD where VFAs accumulates most times due to an imbalance between the acidogenic and methanogenic microbial communities. This among many other factors is responsible for high-level inhibition to digestion and could even cause total system failure. Another major inhibitor to the AD process is the formation of ammonia and its derivatives from nitrogen due to the systematic breakdown of the latter especially at the initial digestion period. However, TN in the fermentation media used in this study was converted and subsequently degraded up to 87% implying a better performance than methanogenesis. As earlier reported, a fuel cell has between 40 to 60% energy efficiency compared with AD with only 19%^65–69^.

Several challenges associated with the use of fuel cell have been evaluated for which solutions have also been propounded^42,51^. However, a major issue is that of lower performance because of low organic content. This can be corrected by using fermentation media that are higher in the concentration of organics such as used in this study i.e. 23.4 and 20.9 g/L which can be increased for better performance of fuel cell. Another challenge is pH fluctuation in the anaerobic fermentation stage and this can be corrected by dosing the medium with weak alkali so as to stabilize the pH at around 6.0–6.5 which is ideal for VFAs formation from substrates^70^. In this study, SAB compared favorably with FW in composition and performance in a fuel cell. Treatment of FW for energy generation is very popular in the energy sector while the SAB generated in most animal rearing systems and experimental laboratories are swept away into the dustbin without any sustainable usage. This study has established the high potential of this bioresource for energy generation, especially in fuel cells. The overall bioelectrochemical performance and comparative electrical recovery from the use of fermentation liquids in the continuous fuel cell’s operation is shown in Table 5. In the first phase, COD removal efficiency reached 78 and 74% for both FW and SAB fermentation liquids respectively while the normalized COD removal rate (NCRR) recorded values of 247.3 and 276 in both FW and SAB respectively. Employing the standard formula, the total electrical energy recovery (*E*_recovered_) from both experiments was 0.2765 and 0.2246 kJ/cm^2^ for FW and SAB respectively. In the second phase, COD removal efficiency reached 56 and 54% respectively for FW and SAB while the NCRR values were 4.6 and 55 for both experiments. The total *E*_recovered_ were 0.1383 and 0.0864 kJ/cm^2^ for FW and SAB respectively. In comparison, better efficiency and higher energy recovery was recorded in the first phase experiments largely due to nutrients abundance.

**Table 5:** Bioelectrochemical performances and energy recovery from the fuel cells during continuous treatment.

Substrate	COD_in_ (g/L)	HRT (h)	ηCOD (%)	NCRR (g/L^−1 ^day^−1^)	*P*_mean_ (mW/cm^2^)	*E*_recovered_ (kJ/cm^2^)
First phase						
FW	15.29	48	78	24.7	1.6	0.2765
SAB	17.91	48	74	27.6	1.3	0.2246
Second phase						
FW	3.94	48	56	4.6	0.8	0.1383
SAB	4.88	48	54	5.5	0.5	0.0864

## Conclusions

It has been shown in this study that a fuel cell can efficiently be used for energy generation from food wastes and spent animal beddings. Anaerobic fermentation of the waste streams was first carried out and the products were very rich in nutrients and volatile fatty acids which were all subsequently and efficiently converted to electricity. The initial extraction of lipids gave the fermentation liquids a better chance of performing as no clogging or inhibition was observed and up to 97% VFAs conversion was achieved. In the same manner, there was high mineralization of carbon to CO_2_ while most nitrogen was degraded to ammonia and subsequently to N_2_. The experiment also showed efficiency in the generation of additional electricity from the effluents in a second treatment with the addition of an external carbon source. Even though food waste has been treated using many technologies in the past, the result of this study showed one of the very best treatment efficiencies achieved for food wastes. Also, spent animal bedding has been shown to be a profound energy resource as it compared favorably well with food waste in this study. Therefore, the use of fuel cell for the treatment of highly volatile waste streams such as different food wastes and others is hereby solicited as this treatment shows higher efficiency than anaerobic digestion at a shorter period.

## Supplementary information

Supplementary Table.
